# Intraoperative Iris Behavior during Phacoemulsification Maneuvers in Rabbits Treated with Selective α1-Blocker, 5α-Reductase Inhibitor, or Anxiolytic Medication

**DOI:** 10.3390/jpm14080840

**Published:** 2024-08-09

**Authors:** Karin Ursula Horvath, Florina Vultur, Septimiu Voidazan, Valentin Simon, Alexandra Cristina Rusu

**Affiliations:** 1 Department of Ophthalmology, George Emil Palade University of Medicine, Pharmacy, Science, and Technology, 540139 Tirgu Mures, Romania; karin.horvath@umfst.ro; 2 Epidemiology Department, George Emil Palade University of Medicine, Pharmacy, Science, and Technology, 540139 Tirgu Mures, Romania; septimiu.voidazan@umfst.ro; 3 Ginecord Clinic SRL, 110132 Pitesti, Romania; valentin_s_ro@yahoo.com; 4 Doctoral School of Medicine and Pharmacy, George Emil Palade University of Medicine, Pharmacy, Science, and Technology, 540139 Tirgu Mures, Romania

**Keywords:** α-blockers, anxiolytics, cataract, 5α-reductase inhibitors, floppy iris

## Abstract

This prospective, experimental study aims to evaluate the association between administration of α-blocker, 5α-reductase inhibitor, or anxiolytic medications and intraoperative floppy iris syndrome (IFIS) using a rabbit animal model. A total of 31 Metis rabbits were distributed into four groups as follows: 10 rabbits given tamsulosin, 10 rabbits given finasteride, 5 rabbits who received lorazepam, and 6 treatment-naive animals in the control group. Dosing was calculated according to body surface area ratio of man to rabbit, with a dosing duration of 43 days for all groups. Phacoemulsification maneuvers were performed by a single surgeon, who was blinded to group allocation. Any intraoperative billowing of the iris was noted and subsequently graded from 0 to 3. Higher incidences of iris billowing were found in the tamsulosin-dosed animals [OR = 8.33 (CI 95% 0.63–110.09)], (*p* = 0.13), the finasteride group [OR = 11.6 (CI 95% 0.92–147.6)], (*p* = 0.11), and the lorazepam group [OR = 7.5 (CI 95% 0.45–122.8)], (*p* = 0.24), as opposed to the control. Administration of α-blocker tamsulosin, 5α-reductase inhibitor finasteride, or anxiolytic medication lorazepam induces altered intraoperative iris behavior. These results correspond with previous studies and further solidify the hypothesis that systemic medication, administered both long and short-term, influences surgical parameters in cataract surgery. The present study can become the basis for further clinical or experimental research.

## 1. Introduction

Two decades after first being mentioned in a paper by Chang and Campbell, intraoperative floppy iris syndrome (IFIS) is now an acknowledged complication of phacoemulsification cataract surgery. Reported as intraoperative iris behavior changes during surgery, IFIS comprises a triad of progressive myosis despite sufficient pharmacologic mydriasis, intraoperative billowing of an atonic iris stroma, and a tendency for iris prolapse towards the phacoemulsification cannula tip and through corneal side-port incisions [1]. Severity-grading systems have been established to describe IFIS, depending on the appearance of signs: mild (only iris billowing), moderate (myosis and iris billowing), and severe (presence of a complete triad) [2].

When IFIS is not anticipated, it can lead to complications such as higher rates of posterior and anterior capsular rupture, residual lens fragments, vitreous loss, iridodialysis, nuclear prolapse, iris trauma, and postoperative atrophy, intraocular pressure elevation (IOP), and cystoid macular edema. These further impact the surgical performance and outcome by prolonging operating room time, associating a higher risk of necessary reintervention, and calling for more complex postoperative treatment regimens. Subsequently, methods of preoperative identification of high-risk patients for anticipating possible IFIS and its complications have become topics of great interest in ophthalmology.

Overall reported prevalence of IFIS ranges between 1.1–12.6%, with several risk factors that have been linked to a higher incidence rate such as age, systemic medications, hypertension, decreased pupil diameter, iris color, and gender [2,3].

Since 1961, when Duke-Elder S. showed that the iris dilator muscle is composed of myoepithelial cells, an interest in iris biomechanics has evolved in ophthalmology. In 1967, Walls GL. described the unique morphologic relationship between pigment epithelium and iris smooth muscle cells influencing drug action [4,5], and Goseki et al. recorded that the high affinity of α-blockers for α1- ARs is an important factor in IFIS development in 2012 [6].

Systemic administration of α1-adrenergic receptor antagonists (α1-blocker/α1-ARA), especially tamsulosin (Tamsol^®^, Omnic tocas^®^, Fokusin^®^, Flomax^®^), is commonly prescribed in benign prostatic hyperplasia (BPH) and lower urinary tract symptoms. BPH affects three out of four males in the seventh decade of life, and treatment with α1-ARAs consistently shows better urinary rates and overall symptom scores. Known as a first-line therapy for BPH patients [7], these medications come with several adverse effects due to the distribution of α1-ARs throughout the body such as dizziness, hypotension, diffuse iris dilator muscle atrophy (through drug–melanin interaction), and IFIS [6].

Because of age group overlap between BPH patients treated with α1-blockers and cataract surgery candidates (68.4 ± 10.2 years) [8], a high percentage of male surgical patients with significant medication history is logically expected. Concurrently, female patients receive selective α1-blockers for urinary retention, and off-label treatment is also used to facilitate passage of urinary stones. A meta-analysis study comparing different systemic α1-ARA effects on the iris has demonstrated that the odds ratio for IFIS after tamsulosin use was about 40-fold higher than for terazosin or alfuzosin, irrespective of treatment duration [3].

Prata et al. demonstrated reduced photopic pupillary diameter in α1-ARA treated patients (2.06 ± 0.5 mm versus 2.5 ± 0.6 mm), alongside significantly lower DMR and DMR/SMR ratio (dilator muscle region and sphincter muscle region thickness) in 63% of treated patients, correlating with the reported incidence of IFIS in patients treated with tamsulosin (62.5% to 93.8%). The rate of DMR/SMR ratio reduction was also positively correlated with α1-ARA treatment duration [9].

Goseki et al. have discovered through electron microscopy that tamsulosin treatment induces vacuolar degeneration of the dilator muscle with the appearance of pigment granules and lobular evolution of dilator muscle cells and pigment epithelial cell nuclei. A later study by the same author on a rabbit animal model investigated α1-ARA drug binding affinity in iris tissues [6].

Alongside α1-ARA-induced IFIS, an entire subset of cases has been attributed to other systemic medications, although the appearance of IFIS might also be correlative or coincidental. Finasteride (Proscar^®^ 5 mg, Propecia^®^ 1 mg) is an inhibitor of type II and III isoenzyme of 5a-reductase enzyme that lowers dihydrotestosterone levels, as used in treatment and control of BPH, to decrease the risk of prostate cancer (with a reduction of 25–30% in men over 55 years of age), to prevent urological events (acute urinary retention), and to treat androgenic alopecia. Although improvement of urologic symptoms occurs after a longer period of treatment compared with α1-AR antagonist medication, finasteride was considered, from an ophthalmologic standpoint, a safer alternative for patients diagnosed with cataracts and BPH. A small number of recent studies have shown a correlation between finasteride treatment, IFIS, and anterior subcapsular cataracts, leading some authors to propose discontinuation before cataract surgery [10,11].

**Figure 1:**
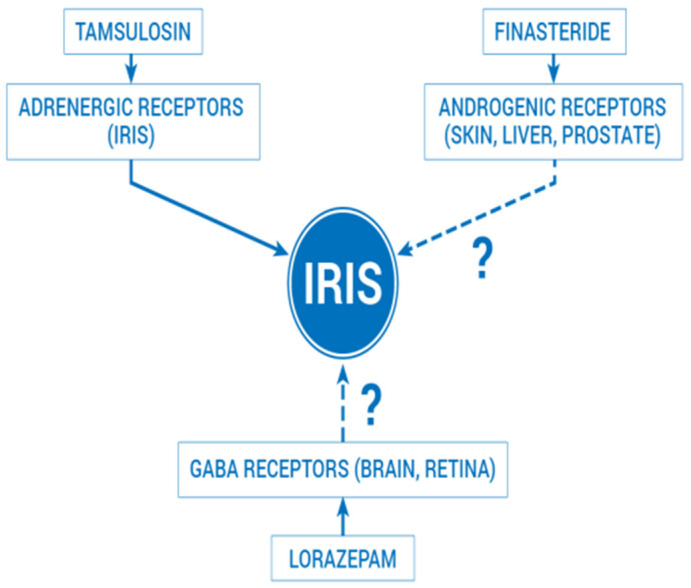
Drug receptor action in medication-induced IFIS (receptors affected by lorazepam and finasteride remain controversial).

A series of neuromodulators from the benzodiazepine class, such as donepezil (acetylcholinesterase inhibitor) and duloxetine (serotonin-norepinephrine reuptake inhibitor SNRI), have also been named possible causative agents of IFIS. Lorazepam (Anxiar^®^ 1 mg) is a benzodiazepine drug that acts on the GABA receptors in the brain and retina (Figure 1) and is used in the treatment of anxiety, somatic disorders, prevention and treatment of delirium tremens, and alcohol withdrawal syndrome [12,13,14,15,16].

This experimental study aimed to evaluate intraoperative iris behavior during simple surgical maneuvers part of cataract extraction through a phacoemulsification protocol in a rabbit model treated with three systemic medications suspected of having correlations with the appearance of IFIS: α1-ARA tamsulosin, 5α-reductase inhibitor finasteride, and the anxiolytic lorazepam.

## 2. Materials and Methods

The current study consists of prospective, in vivo, experimental research on laboratory rabbits housed in the Biobase of the George Emil Palade University of Medicine, Pharmacy, Science, and Technology of Targu Mures, Romania. The full approval of the Ethical Committee was obtained, and steps were taken to avoid animal suffering during the entirety of the experiment. According to the EU Directive 63/2010 “Severity classification of procedures” scale, all chosen surgical and non-surgical maneuvers registered as having moderate-to-low impact on the general health of the animals.

A total of 31 male Metis rabbits, aged 1.5–2 years and weighing between 3.4 and 5.6 kg, were included and distributed in 4 groups as follows: Control Group 1, including 6 treatment-naive animals; Group 2, including 10 rabbits given 0.4 mg/kg tamsulosin 0.4 mg/kg (TAMSOL^®^ 0.4 mg, GEDEON RICHTER, Tirgu Mures, ROMANIA); Group 3, with 10 rabbits given 2.5 mg/kg finasteride (PROSCAR^®^ 5 mg MERCK SHARP & DOHME BV—HOLLAND); and Group 4, consisting of 5 rabbits who received 0.5 mg/kg lorazepam (ANXIAR^®^ 1 mg GEDEON RICHTER ROMANIA).

The drugs were administered through gavage feeding once per day for 43 consecutive days before surgical intervention. Dosage was calculated according to the body surface area ratio of man/rabbit, with respect to the median lethal dose LD50. Tablets were first crushed into powder and then mixed with distilled water 0.8–1.2 mL and emulsifying agent Tween 80 (CRODA INTERNATIONAL PLC SIGMA-ALDRICH^®^ Saint Louis, MO 63103, USA). Exclusion from the study was not necessary during drug administration, the substances being well supported without the need for dose adjustment. No difficulties regarding dosing, management, and upkeep of the laboratory rabbits were encountered.

After randomized animal selection, the surgical maneuvers were performed under general anesthesia by a single surgeon, blinded to group allocation. The chosen anesthesia protocol consisted first of intramuscular administration of 10 mg/kg xylazine (solution 20 mg/mL), succeeded after 10 min by 40 mg/kg ketamine (solution 100 mg/mL), permitting gradual induction over 5–10 min. In all cases, full sedation was obtained after 20 min with the caveat that for the animals given lorazepam a supplementary dose of 20 mg ketamine (0.2 mL) was necessary. The increase in drug resistance was explained by the prior anxiolytic drug regimen. Due to incomplete surgical protocol, 7 out of 62 operated eyes were excluded from the result analysis. Standard preoperative mydriasis with tropicamide was used.

The surgical protocol consisted of the following steps: conjunctival bag instillation of Betadine solution, placement of one 2.2 mm main corneal incision followed by two 1.2 mm side-port incisions, irrigation/aspiration (I/A) maneuvers with bimanual handpieces, and injection of 1 mL physiologic serum using a hydrodisection cannula to induce anterior chamber turbulence. The chosen I/A parameters were a continuous 350 mmHg vacuum, linear 25 mL/min aspiration, and 90 cm maximum perfusion (Alcon Infiniti, Leica M500 microscope). I/A was performed with I/A handpieces, without causing anterior capsular or lens injury and without nucleus phacoemulsification. At the end of the intervention, a topical antibiotic (1 drop VIGAMOX^®^ 5 mg/mL moxifloxacin) was instilled into the conjunctival bag.

Examination of intraoperative iris behavior in the control group allowed baseline parameter identification. Grading of IFIS was performed corresponding to the published literature [17]. All intraoperative billowing of the iris found in the treated animals was noted by the surgeon and one observer and graded from 0 to 3: values greater than or equal to 3 were considered relevant for potential complications with significant change in iris behavior (early stage IFIS), while values from 0 to 2 signified a lack of complications.

Statistical analysis was performed using MedCalc Software (Version 12.3.0 bvba, Marikerke, Belgium) for nominal or quantitative variables. Nominal variables were characterized using frequencies, while quantitative variables were tested for normality of distribution using the Kolmogorov–Smirnov test and were characterized by mean and standard deviation (SD). Student’s *t*-test were used to assess differences between continuous variables, and Fischer’s exact test was used for quantitative variables. A significance level of 0.05 was used for all analyses, and all *p*-values reported are two-tailed.

## 3. Results

ANOVA testing revealed no statistically significant differences between rabbits included in our study regarding mean weight value (*p* = 0.68) (Table 1).

Pupil myosis and iris prolapse (IFIS stages 2 and 3) were absent in all groups during surgical maneuvers.

In the control Group 1, only a few cases with intraoperatively changed iris behavior were recorded (Table 2). From the 12 operated control group eyes, 3 eyes (25.0%) were noted with grade 0 of iris billowing, 3 eyes (25.0%) with grade 1, 5 eyes (41.7%) with grade 2, and one eye (8.3%) was graded 3.

In the tamsulosin-dosed animals (Group 2), 6 eyes were excluded due to incomplete surgical protocol pertaining to rabbit eye morphology. In the 14 remaining eyes, the cannula maneuver induced grade 3 iris billowing in 3 eyes (21.42%) and grade 2 in 5 eyes (35.71%). During the I/A maneuver, grade 2 billowing was recorded in 2 eyes (14.28%) (Table 3). Total grade of recorded iris billowing in Group 2 was 0 in 21.4% (3 eyes), 1 in 21.4% (3 eyes), 3 in 28.6% (4 eyes), 4 in 21.4% (3 eyes), and 5 in 7.1% (one eye).

No eyes from the finasteride-dosed animals in Group 3 were excluded. Cannula irrigation was recorded to induce grade 3 iris billowing in 5 eyes (25%) while grade 2 iris billowing was induced in 4 eyes during the I/A procedure (Table 4). The total grade of recorded iris billowing after finasteride use was 0 in 15.0% (3 eyes), 1 in 15.0% (3 eyes), 2 in 20.0% (4 eyes), 3 in 25.0% (5 eyes), 4 in 5.0% (one eye) and 5 in 20.0% (4 eyes) of Group 3 rabbits.

One eye was excluded from the lorazepam-dosed Group 4 due to incomplete surgical protocol. There was no billowing recorded in 11.1% (one eye), while grade 1 iris billowing was observed in 22.2% (2 eyes), grade 3 in 44.4% (4 eyes), and grade 5 in 22.2% (2 eyes) (Table 5) of Group 4 animals.

There was no statistically significant animal weight variation between the groups included in the study (*p* > 0.05). The results of iris billowing from the medicated Groups 2, 3, and 4 were then compared with those in control Group 1, as shown in Table 6.

The comparison results from Group 2 versus Group 1 reveal that the risk of intraoperative iris billowing was significantly higher in tamsulosin-medicated rabbits, OR = 8.33 (CI 95% 0.63–110.09). The risk of intraoperative iris billowing was also elevated OR = 11.6 (CI 95% 0.92–147.6) in the case of Group 3 versus Group 1, finasteride thus mirroring the effects of tamsulosin. In the case of Group 4 versus Group 1, a higher risk of intraoperative iris billowing was shown to be moderately increased in the lorazepam-medicated rabbits OR = 7.5 (CI 95% 0.45–122.8).

## 4. Discussion

In this study, we have evaluated the suspected relationship between systemic administration of common medications (α1-ARA, a 5α-reductase inhibitor, and an anxiolytic) and IFIS development using a rabbit animal model. While the mechanisms involved in the pathophysiology of IFIS remain unclear, some authors postulate that an increase in liquid outflow from the anterior chamber and through the corneal incisions, high intraocular pressure, variations during cataract surgery, and particular anterior chamber anatomical variations could induce the phenomenon. Subsequently, as soon as IFIS is recognized intraoperatively, it is now advised to decrease liquid inflow (irrigation). Based on this principle, some anatomical variations that lower the anterior chamber’s compliance with fluid inflow while increasing outflow load might also play a role in IFIS through two mechanisms: elevating the pressure gradient across the iris increases fluid outflow, while the iris is brought forward and thus closer to the corneal wounds. This hypothesis indicates that the preoperative study of lens and anterior chamber biomechanics (shallower anterior chamber depth, higher lens vault, thicker lenses, narrow mydriatic pupil) could help predict IFIS risk [2,8,18].

To date, the antagonism between α1-ARAs and α1-receptors found within the dilator muscle cells of the iris inhibiting full dilation during surgery remains the strongest hypothesis. α1-adrenoceptors (α1-ARs) regulate several intra and periocular physiological actions such as ocular vessel tonicity, lacrimal gland secretion of proteins, or pupil diameter. The three subtypes discovered (α1A, α1B, and α1D) are catecholamine-dependent receptors that influence constriction of smooth muscle cells. While α1A-AR is the dominant receptor in the iris dilator muscle mediating contraction, α1B-AR is thought to play an important role in choroidal vessel regulation, and thusly in retinal layer homeostasis and intraocular pressure modulation via vasomotor blood flow control. A study by Dogan et al. highlights the potential effects of α1-ARA use on pupillary diameter and choroidal thickness, postulating possible changes in choroidal morphology and blood supply that require further investigation [19].

Regular discontinuation of α1-ARAs before the intervention, preoperative topical atropine, and low-dose bisulfite-free intracameral epinephrine (theoretically displacing the α1-ARA) may increase iris tone and lower the incidence of IFIS. Unfortunately, some patients with severe cases of IFIS have altered intraoperative iris behavior despite such measures, indicating other unknown mechanisms that act concomitantly [2].

Due to the recognition by the American Society of Cataract and Refractive Surgery and the American Academy of Ophthalmology [20] of the strong correlation between the appearance of IFIS and systemic administration of α1-ARAs, the inclusion of a tamsulosin-dosed group was chosen for this study. In the context of clear documentation regarding α-blocker activity in rabbit iris, the 10 animals given tamsulosin acted as activity witnesses.

**Figure 2:**
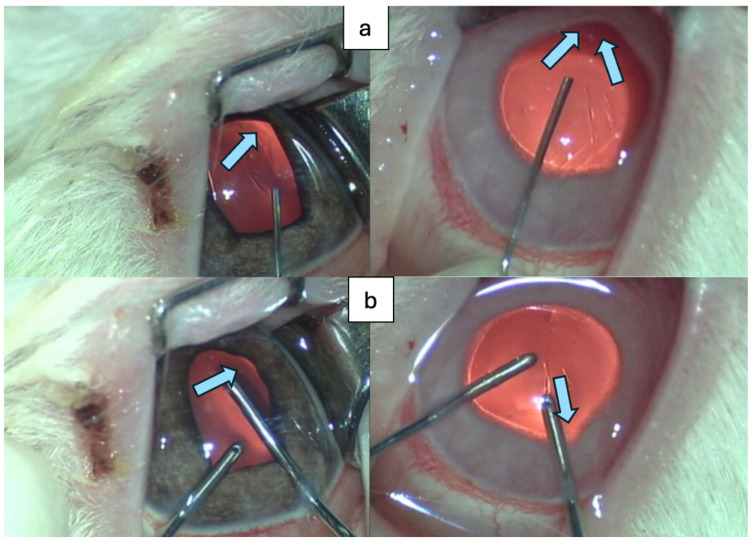
Examples of abnormal iris behavior during surgical protocol on dosed rabbits (direction of iris movement highlighted with arrows): (**a**) cannula BSS injection through a 2.2 mm corneal incision; (**b**) I/A maneuver through 1.2 mm side port incision.

Various mammal species have been used to date in ophthalmology research. While studies on non-human primates are linked to sometimes prohibitive costs, and small rodent models do not permit surgical maneuvers due to reduced eyeball size, the use of rabbits (Oryctolagus cuniculus) overcomes such impediments. Additionally, rabbits share similar heterogeneous genetic backgrounds and physiology with humans, being phylogenetically closer to primates than to rodents. If the anatomical particularities of rabbit eyes such as anterior chamber depth (approximatively 3.1 mm in humans versus 2.9 mm in rabbits), corneal thickness (approximatively 0.2 mm thinner in rabbits), and lens thickness (with an average of 4.0 mm in humans versus 7.9 mm in rabbits) are not omitted, this animal model permits good evaluation of intraoperative behavior, comparable to human eyes, as seen in Figure 2 [21,22].

The four animal groups included in the present study showed average dispersion mass (with a coefficient of variation between 10 and 30%), while the number of rabbits in each group and the duration of drug administration were chosen considering practical limitations such as the total number of animals and drug quantities available.

The iris behavior in tamsulosin-dosed animals showed a higher risk of billowing with an odds ratio of 8.33, despite not inducing any myosis or iris prolapse. Although the reduced sample size did not permit a statistically significant correlation, an impact on the intraoperative iris behavior (iris billowing) during I/A and cannula irrigation maneuvers was observed. This result is in accordance with published data on α1-ARA-induced iris response during cataract surgery [5,23,24].

When compared to the control group, the finasteride-dosed animals also demonstrated an increased rate of iris billowing, with an odds ratio of 11.6. Grade 3 billowing was induced in the same number of eyes in both tamsulosin and finasteride, while maximum grade 5 was comparably higher in the latter (20.0% versus 7.1%). These results, though not proving a statistically significant correlation, do not contradict published data. Multivariate analysis has recently revealed that finasteride use (*p* = 0.014) is strongly associated with IFIS during phacoemulsification [11,25,26,27].

Chronic treatment with lorazepam in different doses often shows up in age groups with a high incidence of cataracts, also frequently appearing in medication histories of patients with IFIS admitted to the Targu Mures Ophthalmology Clinic. The results of the lorazepam-dosed group included in the study, though more modest, revealed an increased ratio of iris billowing, with an associated odds ratio of 7.5.

None of the animals in the study developed moderate (myosis and iris billowing) or severe (presence of a complete triad) IFIS during either the I/A or the cannula irrigation maneuvers.

Though our results are not entirely superposable with the clinical observations on human patients, this experimental study leads to similar conclusions. The results managed to prove that intraoperative iris behavior alterations exist (i.e., early-stage IFIS that may be associated with complications) and occur more often in rabbits treated with the drugs tested.

This study has several limitations. Firstly, due to practical constraints, the chosen animal model was the laboratory rabbit despite non-human primates evidently being the model most capable of mimicking human clinical presentation of ocular biomechanics. Secondly, the duration of drug administration prior to the surgical maneuver was limited to 43 days, which only permits evaluation of the effects of short- to medium-term administration of the chosen drugs. In addition, due to cost limitations, the groups differed in size, which also impacted the final results.

## 5. Conclusions

This experimental, in vivo study on rabbits supports previous clinical observations regarding IFIS and systemic drug administration, and while the results did not show a statistically significant positive correlation due to study limitations (small batch of animals and a restricted drug administration period), they highlighted the impact of pharmacological substances on intraoperative iris behavior (iris billowing) during I/A and cannula irrigation maneuvers.

As a consequence, routine history of 5α-reductase inhibitors and anxiolytics should be adopted alongside α1-ARAs for all patients requiring cataract surgery in order to better anticipate IFIS development and thus reduce possible complications such as higher rates of posterior and anterior capsular rupture, residual lens fragments, vitreous loss, iridodialysis, nuclear prolapse, iris trauma and postoperative atrophy, postoperative intraocular pressure (IOP) elevation, and cystoid macular edema. Anticipating surgical complications will result in more comfort for both the operator, who can take measures to minimize IFIS occurrence, and the patient, who is adequately informed and can better manage their own perioperative expectations.

The results of this study contribute to the growing data on this intraoperative phenomenon, while possibly constituting a basis for future clinical and experimental studies.

In conclusion, the proper identification of IFIS-predisposing factors such as a history of α-blocker, 5α-reductase inhibitor, and anxiolytic drug use is essential for consistent good surgical outcomes, alongside thorough preoperative documentation and in-depth surgeon–patient discussion. Anticipation is crucial in managing aberrant iris behavior during phacoemulsification and for avoiding possible complications that lower the chances of surgical success.

## Figures and Tables

**Table 1:** Mean weight values in the four groups (minimum, median, maximum and mean values expressed in grams).

	Group 1	Group 2	Group 3	Group 4
Number of values	6	10	10	5
Minimum	3.600	3.500	3.500	3.400
Median	4.400	4.100	4.400	4.400
Maximum	5.600	4.800	5.000	4.800
Mean	4.500	4.140	4.240	4.140
Std. Deviation	0.8922	0.4835	0.5038	0.6066

**Table 2:** Distribution of non-treated animals (Group 1) by weight, operated eye, billowing grade during cannula irrigation and irrigation/aspiration.

Animal No.	Weight (kg)	Eye	Iris Behavior Cannula	Iris Behavior I/A	Total	Remarks
C1	5.6	Left	1	0	1	Bilateral complete protocol
		Right	2	1	3	
C2	4.8	Left	0	0	0	Bilateral complete protocol
		Right	0	0	0	
C3	3.6	Left	2	0	2	Bilateral complete protocol
		Right	2	0	2	
C4	5.4	Left	2	0	2	Bilateral complete protocol
		Right	1	0	1	
C5	3.5	Left	1	0	1	Bilateral complete protocol
		Right	0	0	0	
C6	4	Left	1	1	2	Bilateral complete protocol
		Right	1	1	2	

**Table 3:** Distribution of animals treated with tamsulosin (Group 2) by weight, operated eye, billowing grade during cannula irrigation and irrigation/aspiration.

Animal No.	Weight (kg)	Eye	Iris Behavior Cannula	Iris Behavior I/A	Total	Remarks
T1	4.5	Left	0	0	0	Bilateral complete protocol
		Right	0	0	0	
T2	4.4	Left	3	2	5	Bilateral complete protocol
		Right	2	1	3	
T3	4.8	Left	2	1	3	Bilateral complete protocol
		Right	3	1	4	
T4	4.8	Left	2	1	3	Bilateral complete protocol
		Right	2	1	3	
T5	4.2	Left	-	-	-	Bilateral incomplete protocol
		Right	-	-	-	
T6	4	Left	1	0	1	Bilateral complete protocol
		Right	0	0	0	
T7	4	Left	-	-	-	Bilateral incomplete protocol
		Right	-	-	-	
T8	3.5	Left	1	0	1	Bilateral complete protocol
		Right	1	0	1	
T9	3.6	Left	-	-	-	Left eye—incorrect wound
		Right	3	1	4	
T10	3.6	Left	-	-	-	Left eye—incorrect wound
		Right	2	2	4	

**Table 4:** Distribution of animals treated with finasteride (Group 3) by weight, operated eye, and billowing grade during cannula irrigation and irrigation/aspiration.

Animal No.	Weight (kg)	Eye	Iris Behavior—Cannula Maneuver	Iris Behavior—Irrigation Aspiration	Total	Remarks
F1	3.5	Left	1	0	1	Bilateral complete protocol
		Right	2	1	3	
F2	3.8	Left	1	0	1	Bilateral complete protocol
		Right	2	1	3	
F3	4.4	Left	0	0	0	Bilateral complete protocol
		Right	2	1	3	
F4	5	Left	2	0	2	Bilateral complete protocol
		Right	1	0	1	
F5	4.2	Left	3	2	5	Bilateral complete protocol
		Right	3	2	5	
F6	4.8	Left	3	2	5	Bilateral complete protocol
		Right	3	2	5	
F7	4.4	Left	2	1	3	Bilateral complete protocol
		Right	1	1	2	
F8	3.5	Left	1	1	2	Bilateral complete protocol
		Right	1	1	2	
F9	4.4	Left	2	1	3	Bilateral complete protocol
		Right	3	1	4	
F10	4.4	Left	0	0	0	Increased dosage of anesthetic drug
		Right	0	0	0	

**Table 5:** Distribution of animals treated with lorazepam (Group 4) by weight, operated eye, and billowing grade during cannula irrigation and irrigation/aspiration.

Animal No.	Weight (kg)	Eye	Iris Behavior—Cannula Maneuver	Iris Behavior—Irrigation Aspiration	Total	Remarks
A1	3.4	Left	2	1	3	Bilateral complete protocol
		Right	2	1	3	
A2	4.4	Left	1	0	1	Bilateral complete protocol
		Right	1	0	1	
A3	4.8	Left	2	1	3	Increased dosage of anesthetic drug
		Right	2	1	3	
A4	3.6	Left	-	-	-	Bilateral complete protocol
		Right	0	0	0	
A5	4.5	Left	3	2	5	Bilateral complete protocol
		Right	3	2	5	

**Table 6:** Comparison between treated and non-treated animals.

Medication Risk Factor (≥3)	Potential to Induce Complications			OR (CI: 95%)	*p* Value
	Present	Absent	Total		
Tamsulosin	5	3	8	8.33 (0.63–110.09)	0.13 ^1^
Finasteride	7	3	10	11.6 (0.92–147.6)	0.11 ^2^
Lorazepam	3	2	5	7.5 (0.45–122.8)	0.24 ^3^
Control	1	5	6	-	-

^1^ Fisher’s comparison between Group 1—control and Group 2—tamsulosin; ^2^ Fisher’s comparison between Group 1—control and Group 3—finasteride; ^3^ Fisher’s comparison between Group 1—control and Group 4—lorazepam.

## Data Availability

The original contributions presented in the study are included in the article; further inquiries can be directed to the corresponding author.
